# Agnostic Envelope Linearization of Dynamically Supplied Power Amplifiers for Mobile Terminals

**DOI:** 10.3390/s22103773

**Published:** 2022-05-16

**Authors:** Wantao Li, Gabriel Montoro, Pere L. Gilabert

**Affiliations:** Department of Signal Theory and Communications, Universitat Politècnica de Catalunya (UPC), Barcelona Tech., 08034 Barcelona, Spain; wantao.li@upc.edu (W.L.); gabriel.montoro@upc.edu (G.M.)

**Keywords:** digital predistortion, envelope tracking, power amplifier, RF leakage

## Abstract

This paper presents an envelope linearization technique to compensate for the nonlinear distortion of envelope tracking (ET) power amplifiers (PAs) for 5G new radio (NR) mobile terminals. The proposed envelope optimization (EOPT) method is agnostic of the nonlinear distortion generated in the envelope supply path and can compensate for the nonlinear distortion at the ET PA output without the need to monitor the output at the envelope tracking modulator (ETM). The linearization system in the envelope path is based on the envelope generalized memory polynomial (EGMP) behavioral model. Since the ETM output is not available, an iterative nonlinear least squares solution inspired in the deep deterministic policy gradient (DDPG) algorithm is proposed to extract the coefficients of the EGMP model. The EOPT method is validated on a system-on-chip (SoC) ET PA board designed for mobile terminal applications. Experimental results show the suitability of the proposed method to guarantee the linearity requirements (i.e., adjacent channel power ratio below −36 dBc) with 16.8% of power efficiency when operating the ET PA with 5G new radio test signals of 60 MHz bandwidth operating at 2.55 GHz (band 7). The linearization performance of the proposed EOPT method is comparable to the envelope leakage cancellation (ELC) approach (but saving the need for an analog to digital converter to monitor the ETM output), and can outperform a conventional I-Q digital predistorter based on the generalized memory polynomial (GMP) behavioral model.

## 1. Introduction

Power efficient and linear amplification of radio frequency (RF) power amplifiers (PAs) is an old but still hot topic of research, since with the eruption of new technologies (in 4G, 5G, and beyond), new challenges arise in terms of required transmit power, transmission, and reception with multiple antennas, dynamic bandwidth allocation (i.e., numerology), dynamic or concurrent frequency bands of operation, and high peak-to-average power ratio (PAPR) waveforms as a consequence of targeting spectrally efficient high-capacity transmission (i.e., high-order M-QAM modulation schemes and orthogonal frequency-division multiplexing—OFDM).

Several adaptive digital predistortion (DPD) schemes have been proposed to linearize RF PAs in macro base stations [1,2], where the DC power consumption of the RF PA is significantly higher than the DC power consumption of the digital signal processing (DSP) hardware. However, the linearization of the user equipment (UE) requires low-complexity and, preferably, open-loop linearization solutions, since the DC power consumption of the digital signal processing (DSP) significantly impacts the overall transmitter power consumption [3,4,5].

In addition, many efforts have been devoted to find highly efficient amplification topologies capable of maintaining high power efficiency figures over significant back-off levels [6]. Power amplifier architectures based on dynamic load modulation, such as Doherty PAs [7], load modulated balanced amplifiers (LMBA) [8], or outphasing or LINC PAs [9], have been widely proposed in the literature. Moreover, dynamic supply modulation techniques, such as envelope tracking (ET) PAs [10,11,12], have been adopted in commercial solutions for mobile terminals in order to mitigate the DC power dissipated as heat when handling signals with high PAPR.

The overall power efficiency in ET PAs results from the product of the RF PA drain power efficiency times the efficiency of the envelope tracking modulator (ETM). The ETM is responsible for delivering the instantaneous power required by the RF PA; therefore, its design is key to determine the trade-off between linearity and power efficiency in ET PAs. Typically, a linear-assisted hybrid configuration [13,14,15,16] is adopted in the design of ETMs.

When dealing with wideband envelope signals (according to the rule of thumb, the signal’s envelope is around three to five times the bandwidth of the complex baseband signal), the ETM performance in terms of linearity and power efficiency is degraded. A solution to cope with the aforementioned trade-off is to generate a slower (i.e., bandwidth or slew-rate limited) version of the original supply envelope through a specific shaping function [17,18]. In addition, as reported in [19], unwanted nonlinear distortion in the envelope path may arise due to the intrinsic topology of the hybrid ETM when amplifying wideband signals. In particular, the coupling between the ETM and the RF PA is not ideal (more evident at higher frequencies), and thus, due to this load effect, the RF leakage cannot be eliminated or absorbed by the ETM and it is combined with the supply voltage, degrading the overall linearity at the RF PA output.

An envelope leakage cancellation (ELC) method was proposed by the authors of this paper in [19] to linearize the unwanted RF leakage and nonlinear distortion effects when operating the ET PA with a 60 MHz bandwidth 5G NR test signal. The ELC method relied on the envelope generalized memory polynomial (EGMP) behavioral model to generate a cancellation signal capable of compensating for the unwanted RF leakage in the dynamic supply path. Unfortunately, this solution assumes that the output of the ETM is available through a dedicated observation path (including an analog-to-digital converter—ADC—with sampling rate at least 6× the I-Q signal bandwidth) to estimate the leakage error. However, having access to the ETM output is not always possible (e.g., this external port is not included for better and easier impedance matching) and would require an extra ADC to capture the supply envelope after amplification. As mentioned before, low-complexity and robust open-loop solutions are preferred in the UE, since the impact of the DSP consumption is no longer negligible.

In this context, as an alternative to the ELC method in [19], an envelope optimization (EOPT) method is proposed in this paper to linearize the ET PA RF output without the need for monitoring the ETM output. Thus, saving the cost of including an extra ADC and a matching transformer (in between the ETM output and the ADC). The proposed EOPT method is also based on the EGMP behavioral model. However, the offline identification procedure to estimate the coefficients of the EGMP model is completely different from [19]. The identification process is inspired by the deep deterministic policy gradient (DDPG) algorithm, a popular algorithm for reinforcement learning [20]. In addition, I-Q DPD based on the generalized memory polynomial (GMP) behavioral model is also considered for ET PA linearization to better highlight the contribution of the proposed envelope optimization method. Experimental results will show a comparison of the proposed EOPT method with both the ELC approach in [19] and the conventional I-Q DPD in terms of linearization performance and computational cost.

The remainder of this paper is organized as follows. A detailed explanation of the proposed EOPT approach for wideband ET PAs is given in Section 2, including an in-depth description of the main key subsystems contributing to define the power efficiency and linearity trade-off in ET PAs. The proposed envelope optimization approach is validated on an SoC ET PA board, considering an NR-60 MHz OFDM-like test signal. Thus, experimental results are shown in Section 3, comparing the linearization performance of the proposed EOPT method, which is agnostic of the ETM behavior, with the ELC or I-Q DPD linearization methods. Finally, a conclusion is given in Section 4.

## 2. Envelope Tracking PA Linearization

**Figure 1:**
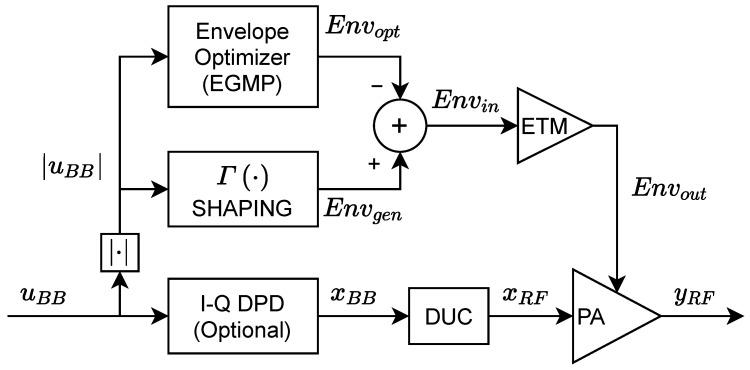
Block diagram of the forward path for ET PA linearization.

The proposed ET PA digital linearization approach that relies only on the observation of the RF PA output is presented in this section. Figure 1 shows the block diagram of the forward path for the proposed ET PA linearization system. It is composed of three major blocks: (i) a shaping function for the generation of the supply envelope; (ii) an envelope optimizer in the supply path that contributes to the ET PA linearization; and (iii) an optional I-Q digital predistorter that can be used if further linearization is required. The shaping function generates the original supply envelope $Env_{gen}$ which determines the efficiency and initial linearity of the ET PA system. The envelope optimizer takes the baseband I-Q input signal envelope (i.e., $|u_{BB}|$) and generates the optimized supply envelope $Env_{opt}$, which, properly combined with $Env_{gen}$, results in the supply envelope $Env_{in}$, oriented at improving the linearity of the ET PA. An I-Q DPD can be included to further enhance the linearity of the ET PA.

In the following subsections, the linearity versus efficiency trade-off in the shaping function responsible for the generation of the supply envelope is first introduced. Then, for the sake of completeness, the EGMP model proposed in [19] and the direct learning approach to extract the EGMP coefficients, assuming that ETM output is available, is presented. Next, to generate the optimized supply envelope $Env_{opt}$ without having access to the ETM output, a slow envelope-dependent (SED) PA behavioral model is used, and an exploration scheme is proposed to help the PA model converge to a global optimal. Finally, the procedure to extract the coefficients of the EGMP model through the SED PA behavioral model is explained. It is important to note that the proposed EOPT method is agnostic on the ETM behavior (i.e., the access to the ETM output is not required) and, thus, it only requires monitoring the RF PA output and a precise ET PA behavioral model to extract the coefficients of the EGMP model.

### 2.1. Supply Envelope Generation and ET PA Nonlinear Behavior

By means of a shaping function, it is possible to accommodate the shape of the supply voltage (that somehow must follow the instantaneous RF envelope) to achieve one of the following objectives: optimum efficiency shaping or isogain shaping [21] (i.e., sacrificing power efficiency for linearity). In addition, in order to deal with the bandwidth limitation of the ETM, the use of a slower version of the supply envelope has been proposed in [17,18], to mitigate the trade-off between power efficiency and linearity.

In general, the generated supply envelope ${Env}_{gen}\left[n\right]$ is defined as

(1) $${Env}_{gen}\left[n\right]=\Gamma \left(|u_{\mathrm{BB}}\left[n\right]|\right)$$

where Γ(·) denotes the shaping function and $u_{\mathrm{BB}}\left[n\right]$ is the complex I-Q baseband signal to be transmitted (see notation in Figure 1). One commonly used memoryless parametric shaping function [22] to accommodate the supply levels and determining the level of detroughing is

(2) $$Env_{\mathrm{DT}}\left[n\right]={\left({\left(E_{\mathrm{TH}}\right)}^{p}+|u_{\mathrm{BB}}\left[n\right]{|}^{p}\right)}^{1/p}$$

where $E_{\mathrm{TH}}$ is the threshold or lower bound of the envelope that determines the swing voltages or dynamic range of the ETM, and *p* is the order of this detroughing function. The offset percentage (OP) of the supply envelope voltage is defined as

(3) $$\mathrm{OP}\;\left(\%\right)=\frac{E_{\mathrm{TH}}}{\max\left(|u_{BB}\left[n\right]|\right)}\cdot 100$$

As observed in (2) and (3), the detroughing function has two degrees of freedom (i.e., OP and *p*) to trade-off the power efficiency and linearity in ET PA.

**Figure 2:**
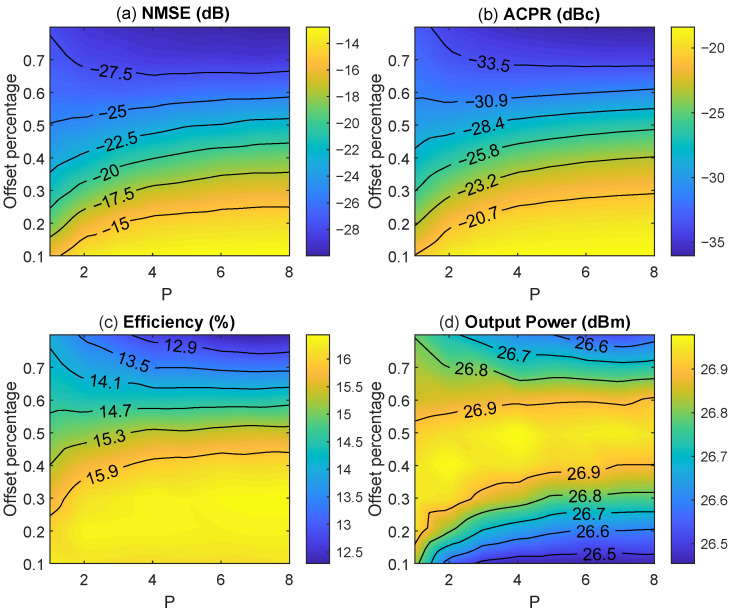
NMSE (**a**), ACPR (**b**), efficiency (**c**), and output power (**d**) performance of the ET PA with the NR-60 MHz OFDM-like signal, using the detroughing envelope with different offset percentage and p configurations.

Figure 2 shows the evolution of key performance indicators such as the adjacent channel power ratio (ACPR), the normalized mean square error (NMSE), the drain power efficiency, and the output power for different configurations of the OP and *p* parameters when considering an NR-60 MHz OFDM-like test signal. As observed in Figure 2, the offset percentage is a key parameter that significantly impacts both the linearity (in terms of NMSE and ACPR) and the drain power efficiency. The higher the OP, the better the linearity but the lower the drain power efficiency. The efficiency can reach 16% when the OP is 0.1, which has the worst ACPR of around −20.7 dBc. When the OP=0.8 and p=8, the ACPR can reach −35 dBc but the efficiency drops to around 13%.

As mentioned before, when dealing with wideband signals, the ETM unwanted effects (i.e., nonlinear behavior, RF leakage) may worsen and the supply envelope linear amplification may be compromised. Consequently, it is possible to include, on top of the detroughing function, a slew-rate reduction (SR) algorithm to cope with the bandwidth limitations of the ETM. The SR reduction algorithm proposed in [18] is described as follows:  

(4) $$Env_{SR}\left[n\right]=\max_{i=0,1,...,N}\left(Env_{DT}\left[n+i\right]-i\cdot \frac{V_{\max}}{N}\right)$$

where N is the number of steps, and $V_{\max}$ is the maximum numerical value of the envelope. In practice, the baseband input is normalized by a scaling factor so that it has a maximum amplitude of 1 ($V_{\max}=1$). The only configuration that needs to be determined for the SR algorithm is the number of steps N. The higher the number of steps, the smaller the slew-rate and the bandwidth of the envelope signal. The SR shaping function is a memory-based algorithm with memory depth N+1 and the hardware implementation cost of the SR algorithm increases with N.

**Figure 3:**
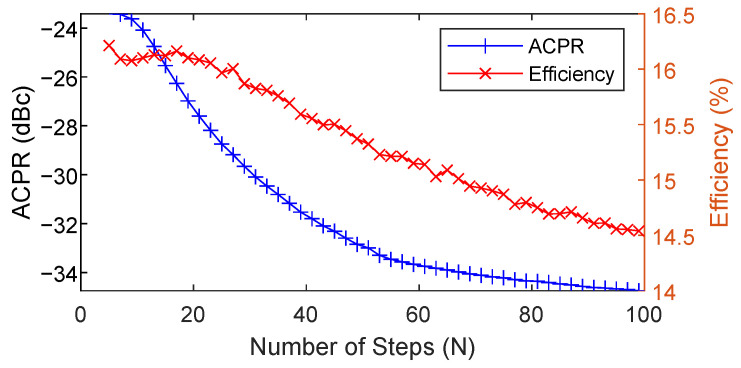
ACPR and efficiency performance of the ET PA with the NR-60 MHz OFDM-like signal, using the SR envelope with p = 1, OP = 0.2, and different N (number of steps) configurations.

Figure 3 shows the ACPR and efficiency performance of the SR envelope with different configuration of N. The SR process is applied on top of the detroughed envelope with a given configuration of OP=20% and p=1, corresponding to an original ACPR around −24 dBc and drain power efficiency around 16%. As observed, the ACPR improves when increasing the number of steps N. The drain power efficiency, however, is only maintained for N<20 and then decreases when increasing N. A good trade-off can be found for N=30, where the drain efficiency is around 15.9% and the ACPR is around −30 dBc.

**Figure 4:**
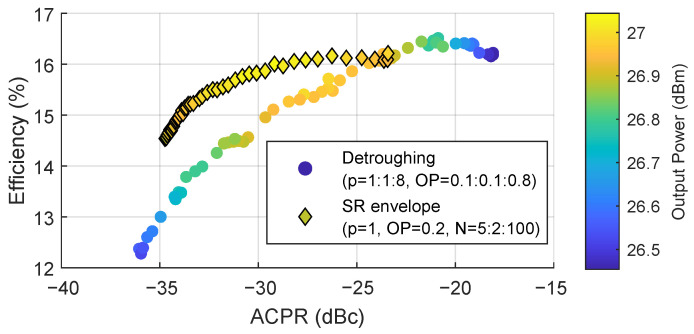
ACPR and efficiency trade-off and comparison between the detroughed envelope and the SR envelope.

Figure 4 shows the scatter plot of the drain power efficiency and ACPR for both the detroughed envelope and the SR reduced envelope, using all the searching test results from Figure 2 and Figure 3. Therefore, Figure 4 shows the power efficiency and linearity trade-off regardless of the envelope generation configuration. Comparing the results obtained with the SR reduced envelope and the detroughed envelope, when the power efficiency is similar (e.g., power efficiency around 16%), the ACPR performance using the detroughed envelope is only −25 dBc. Instead, by using the SR reduced envelope, it is possible to reach −30 dBc of ACPR. Therefore, with the SR reduction algorithm, it is possible to improve the ET PA linearity at the price of degrading the power efficiency. As observed, the number of steps N can be properly chosen to avoid a significant degradation of the drain power efficiency.

**Figure 5:**
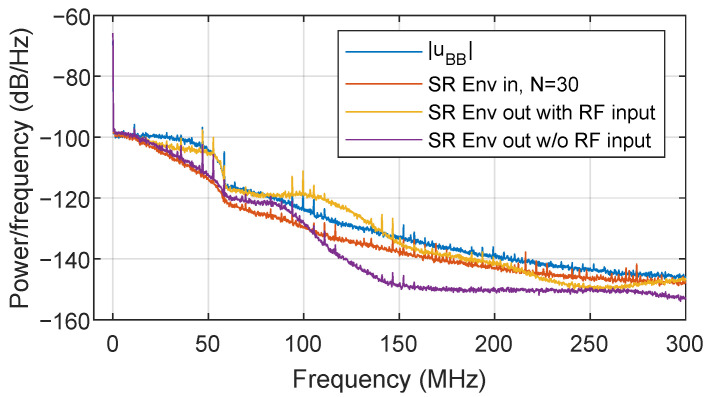
Power spectra density plot of the ETM output of an SR reduced envelope with and without RF input.

However, even after SR reduction of the supply envelope, the ACPR requirement of −36 dBc is still not met, and thus further linearity enhancement is required. As discussed in [19], another source of nonlinear distortion of the ET PA system is the RF to envelope leakage. Due to the intrinsic design of the ETM, the ETM output impedance is not a perfectly constant low value. As a consequence, the distortion at the RF PA output is leaked into the envelope supply path (i.e., the output port of the ETM). This leakage is combined with the supply envelope, causing nonlinear distortion at the RF PA output. Figure 5 shows the spectra of the supply envelope at the ETM output when considering the amplification of an SR reduced envelope (OP=0.2,p=1,N=30). When there is no RF input signal, the ETM can amplify the SR reduced envelope up to its bandwidth limitation. Instead, when the RF input is turned on, some RF leakage is added to the ETM output spectrum, as observed in Figure 5. In the latter case, the spectrum of the amplified supply envelope presents a similar shape to the spectra of the original envelope of the baseband input $|u_{BB}|$. The envelope linearization method proposed in this paper to meet the required linearity specifications is described in the following subsection.

### 2.2. Optimized Envelope Generation for ET PA Linearization

**Figure 6:**
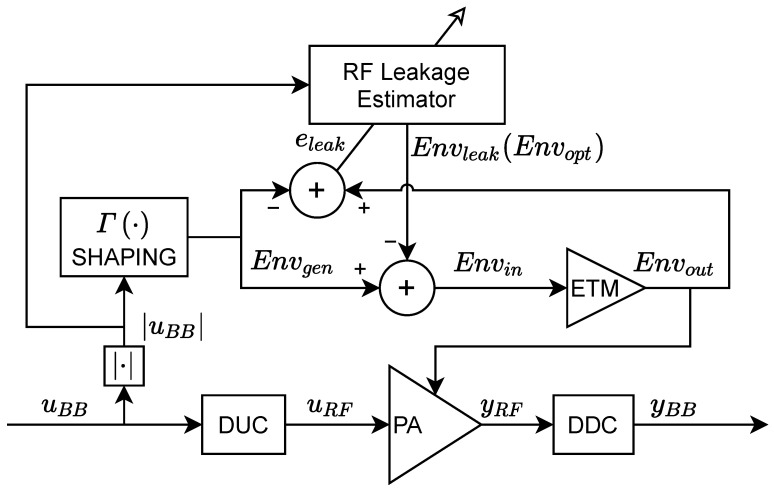
Block diagram of the RF leakage compensation and its coefficient identification.

The proposed linearization method is applied in the supply envelope path. However, unlike in [19], where the ETM output was available to train an ELC subsystem, now it is assumed that the ETM output is no longer accessible. Therefore, instead of targeting the leakage cancellation at the ETM output following the ELC approach (as depicted in the block diagram of Figure 6), an envelope optimizer targeting the linearization of the RF PA output is now proposed (see Figure 1). The envelope optimizer presented in this paper is also based on the generalized memory polynomial (EGMP) model, defined as follows:

(5) $$\begin{array}{c} Env_{\mathrm{opt}}\left[n\right]=\sum_{q=0}^{Q_{a}}\sum_{r=0}^{R_{a}}\sum_{p=0}^{P_{a}}\alpha_{prq}\;|u_{\mathrm{BB}}\left[n-\tau_{r}\right]||u_{\mathrm{BB}}\left[n-\tau_{r}-\tau_{q}\right]{|}^{p} \end{array}$$

where $\tau_{r}$ and $\tau_{q}$ (with $\tau_{r,q}\in Z$) are the most significant sparse delays of the envelope ($|u_{\mathrm{BB}}\left[n\right]|$) that contribute to characterize memory effects, and $\alpha_{prq}$ are the model coefficients.

Following the notation in Figure 1 and  Figure 6, the linearized input envelope to the ETM can be defined in a compact matrix notation as

(6) $$\begin{array}{c} {\mathrm{Env}}_{\mathrm{in}}={\mathrm{Env}}_{\mathrm{gen}}-{\mathrm{Env}}_{\mathrm{opt}}={\mathrm{Env}}_{\mathrm{gen}}-E\;\alpha \end{array}$$

where ${\mathrm{Env}}_{\mathrm{gen}}$ and ${\mathrm{Env}}_{\mathrm{in}}$ are the N×1 vectors of the original supply envelope and the ETM input envelope, respectively, while $E={\left(\vartheta [0],\cdots ,\vartheta [n],\cdots ,\vartheta [N-1]\right)}^{T}$ is the $N\times O_{a}$ EGMP data matrix, with *N* being the number of samples and $O_{a}=\left(Q_{a}+1\right)\left(R_{a}+1\right)\left(P_{a}+1\right)$ the order of the EGMP model. The vector containing the basis functions $\vartheta_{prq}\left[n\right]$ following the EGMP behavioral model described in (5) is $\vartheta^{T}\left[n\right]=\left(\vartheta_{000}\left[n\right],\cdots ,\vartheta_{prq}\left[n\right],\cdots ,\vartheta_{P_{a}R_{a}Q_{a}}\left[n\right]\right)$, while $\alpha^{T}=\left(\alpha_{000},\cdots ,\alpha_{prq},\cdots ,\alpha_{P_{a}R_{a}Q_{a}}\right)$ is the $O_{a}\times 1$ vector of coefficients. As detailed in [23], feature selection techniques can be applied to select the most relevant basis functions and thus reduce the order of the original EGMP behavioral model.

When the ETM output is available, the EGMP model can be trained to predict the RF leakage at the ETM output. Then, this RF leakage estimation is injected in counter-phase to the envelope input to perform the leakage cancellation at the ETM output. For the sake of completeness, Figure 6 shows the block diagram of the RF leakage compensation approach proposed in [19], which uses the leakage estimation $Env_{\mathrm{leak}}$, instead of $Env_{\mathrm{opt}}$ in (6). The leakage is extracted by subtracting the ETM output measurement with the original generated envelope as

(7) $$E_{leak}={\mathrm{Env}}_{out}-{\mathrm{Env}}_{gen}$$

where $e_{\mathrm{leak}}={\left(e_{leak}\left[0\right],\cdots ,e_{leak}\left[n\right],\cdots ,e_{leak}\left[N-1\right]\right)}^{T}$ is the N×1 error vector. Then, the EGMP model coefficients can be extracted iteratively finding the least squares (LS) solution. At the ith iteration it is described as follows:

(8) $$\alpha^{i+1}=\alpha^{i}+\mu {\left(E^{T}E\right)}^{-1}E^{T}e_{leak}$$

As shown in [19], by canceling the RF leakage at the ETM output, the overall linearity of the ET PA system is improved without impacting the efficiency of the ET PA.

In a more realistic and low-cost (i.e., an extra ADC is saved) scenario in which the ETM output measurement is not accessible, there is no reference to directly train the EGMP model for estimating the unwanted RF leakage. However, having the empirical evidence of the capabilities of the EGMP model to compensate for the RF leakage and linearize the ET PA, it is fair to say that the EGMP model can generate an optimized envelope signal that contributes to the linearization of the RF signal at the ET PA output.

**Figure 7:**
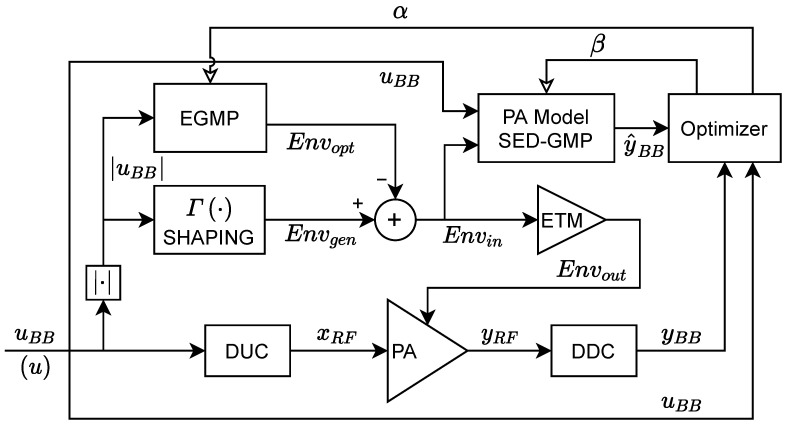
Block diagram of the optimized envelope generation scheme, the measurement of ${\mathrm{Env}}_{out}$ is not required by the optimizer.

In this context, the goal of this paper is to extract the EGMP model coefficients to generate an optimized envelope that contributes to the linearization of the RF signal at the ET PA output. Since the RF output of the ET PA is always available for offline training, it is possible to model/predict the ET PA output given the baseband I-Q input and the supply envelope. Figure 7 shows the block diagram of the proposed optimized envelope generation scheme. Unlike the ELC method, the proposed envelope optimizer requires only monitoring the ET PA output to build a supply-envelope-dependent PA behavioral model. Then, it uses the ET PA behavioral model to train the coefficients of the EGMP model and generate the optimized envelope in (6). The supply-envelope-dependent ET PA model is presented in the following subsection.

### 2.3. Supply-Envelope-Dependent ET PA Modeling

In order to model the ET PA output taking into account the supply envelope ($Env_{\mathrm{in}}\left[n\right]$) and baseband I-Q input signal ($u_{\mathrm{BB}}\left[n\right]$), a two-dimensional supply-envelope-dependent generalized memory polynomial (SED-GMP) behavioral model is proposed. The SED-GMP is composed of two parts, the ordinary GMP part that contains nonlinear and memory polynomial terms of the input signal envelope, $|u_{\mathrm{BB}}\left[n\right]|$, to model the PA nonlinear behavior; and the SED part that includes the nonlinear and memory polynomial terms of the supply envelope, $Env_{in}\left[n\right]$, to characterize the ETM unwanted behavior. The SED-GMP input output relationship is defined as follows:

(9) $$\begin{array}{c} {\hat{y}}_{\mathrm{BB}}\left[n\right]=\sum_{q=0}^{Q_{b}}\sum_{r=0}^{R_{b}}\sum_{p=0}^{P_{b}}\beta_{prq}^{u}\;u_{\mathrm{BB}}\left[n-\tau_{r}\right]{\left|u_{\mathrm{BB}}\left[n-\tau_{r}-\tau_{q}\right]\right|}^{p}\\ +\sum_{s=0}^{S_{b}}\sum_{l=0}^{L_{b}}\sum_{m=0}^{M_{b}}\beta_{mls}^{e}\;u_{\mathrm{BB}}\left[n-\tau_{l}\right]{\left(Env_{in}\left[n-\tau_{l}-\tau_{s}\right]\right)}^{m} \end{array}$$

where $\tau_{r}$, $\tau_{q}$, $\tau_{l}$, and $\tau_{s}$ (with $\tau_{r,q,l,s}\in Z$) are the most significant sparse delays of the baseband I-Q input signal ($u_{\mathrm{BB}}\left[n\right]$), its envelope ($|u_{\mathrm{BB}}\left[n\right]|$) and the input supply envelope ($Env_{in}\left[n\right]$) that contribute to characterize memory effects; $\beta_{prq}^{u}$ and $\beta_{mls}^{e}$ are the coefficients of the GMP and SED parts, respectively. The order of the SED-GMP behavioral model is $O_{b}=\left(Q_{b}+1\right)\left(R_{b}+1\right)\left(P_{b}+1\right)+\left(S_{b}+1\right)\left(L_{b}+1\right)\left(M_{b}+1\right)$.

Similar to (6), the ET PA output estimation can be rewritten in a matrix notation as

(10) $$\begin{array}{cc} {\hat{y}}_{\mathrm{BB}} & =U\beta \end{array}$$

where ${\hat{y}}_{\mathrm{BB}}$ is the N×1 vector of the estimated baseband ET PA output, $U=\left(U_{u}\;\;U_{e}\right)={\left(\phi [0],\cdots ,\phi [n],\cdots ,\phi [N-1]\right)}^{T}$ is the $N\times O_{b}$ SED-GMP data matrix, with *N* being the number of samples and $O_{b}$ the order of the SED-GMP model, and $U_{u}$ and $U_{e}$ are the data matrices of the GMP and SED parts in (9), respectively. The vector containing the basis functions following the SED-GMP behavioral model described in (9) is $\phi^{T}\left[n\right]=\left(\phi_{000}^{u}\left[n\right],\cdots ,\phi_{prq}^{u}\left[n\right],\cdots ,\phi_{P_{b}R_{b}Q_{b}}^{u}\left[n\right],\phi_{000}^{e}\left[n\right],\cdots ,\phi_{mls}^{e}\left[n\right],\cdots ,\phi_{M_{b}L_{b}S_{b}}^{e}\left[n\right]\right)$, while the $O_{b}\times 1$ vector $\beta^{T}=\left(\beta^{u}\;\;\beta^{e}\right)=\left(\beta_{000}^{u},\cdots ,\beta_{prq}^{u},\cdots ,\beta_{P_{b}R_{b}Q_{b}}^{u},\beta_{000}^{e},\cdots ,\beta_{mls}^{e},\cdots ,\beta_{M_{b}L_{b}S_{b}}\right)$ is the vector of coefficients.

After measuring, time-aligning and normalizing (by the targeted linear gain $G_{0}$) the ET PA output $y_{\mathrm{BB}}$, the coefficients of the SED-GMP model can be extracted by means of the ordinary LS estimation as follows:

(11) $$\beta^{\prime }={\left(U^{H}U\right)}^{-1}U^{H}y_{\mathrm{BB}}$$

where the superscript *H* stands for the Hermitian transpose. After a first coefficient identification, the coefficients of the SED-GMP model can be iteratively updated using new input and output ET PA measurements as follows:

(12) $$\beta^{i+1}=\gamma \beta^{\prime }+\left(1-\gamma \right)\beta^{i}$$

where $\beta^{i}$ is the SED-GMP coefficients at *i*th iteration, $\beta^{\prime }$ is the vector of coefficients extracted taking into account the latest data measurements as described in (11), and γ (0<γ<1) is the learning rate. With this SED-GMP model, it is possible to predict the output and estimate the linearity of the ET PA under a specific supply envelope. In the next subsection, the coefficients adaptation procedure for the EGMP model is described by exploiting the proposed SED-GMP behavioral model.

### 2.4. EGMP Coefficients Extraction Process

In order to extract the coefficients of the EGMP behavioral model that contribute to obtain the required linearity at the RF ET PA output, the goal of minimizing the mean square error between the ET PA output estimation, ${\hat{y}}_{\mathrm{BB}}$, and the baseband input, $u_{\mathrm{BB}}$, is defined as follows:

(13) $$\alpha_{opt}=\arg\min_{\alpha }{∥u_{\mathrm{BB}}-{\hat{y}}_{\mathrm{BB}}∥}^{2}=\arg\min_{\alpha }{∥e_{\mathrm{BB}}∥}^{2}$$

with $\alpha_{opt}$ being the vector of optimum coefficients that minimize the mean square error. Since the supply envelope ($Env_{in}\left[n\right]$) is part of a nonlinear polynomial expansion in (9), a nonlinear least squares solution is proposed to address this minimization problem. Instead of numerical differentiation, in order to improve the adaptation accuracy and reduce the calculation time, analytical differentiation is used in this paper. The analytical solution requires the Jacobian matrix being provided to the solver. The Jacobian of the SED-GMP model to the coefficients of the EGMP model is

(14) $$J=\left(\frac{\partial {\hat{y}}_{\mathrm{BB}}}{\partial \alpha_{000}},\cdots ,\frac{\partial {\hat{y}}_{\mathrm{BB}}}{\partial \alpha_{prq}},\cdots ,\frac{\partial {\hat{y}}_{\mathrm{BB}}}{\partial \alpha_{P_{a}R_{a}Q_{a}}}\right)$$

with α being the $O_{a}\times 1$ vector of coefficients of the EGMP model in (5) and (6) and ${\hat{y}}_{\mathrm{BB}}$ being the estimated ET PA output in (9) and (10). The ordinary GMP part in (9) can be ignored in the differentiation. Therefore, by selecting the terms in (9) involving the supply envelope ($Env_{in}\left[n\right]$) and the coefficients $\beta_{mls}^{e}$, it is possible to define the following basis functions:

(15) $$f_{mls}\left[n\right]=\beta_{mls}^{e}\;u_{\mathrm{BB}}\left[n-\tau_{l}\right]{\left(Env_{in}\left[n-\tau_{l}-\tau_{s}\right]\right)}^{m}$$

The Jacobian of the basis functions $f_{\mathrm{mls}}$ can be derived by applying the chain rule as follows:

(16) $$\begin{array}{c} J_{f_{\mathrm{mls}}}=\left(\frac{\partial f_{\mathrm{mls}}}{\partial \alpha_{000}},\cdots ,\frac{\partial f_{\mathrm{mls}}}{\partial \alpha_{prq}},\cdots ,\frac{\partial f_{\mathrm{mls}}}{\partial \alpha_{P_{a}R_{a}Q_{a}}}\right) \end{array}$$

(17) $$J_{f_{\mathrm{mls}}}\left[n\right]=\beta_{mls}^{e}\;u\left[n-\tau_{l}\right]\;{\left(Env_{in}\left[n-\tau_{l}-\tau_{s}\right]\right)}^{m-1}\vartheta [n-\tau_{l}-\tau_{s}]\;m$$

where $\vartheta \left[n\right]=\left(\vartheta_{000}\left[n\right],\cdots ,\vartheta_{prq}\left[n\right],\cdots ,\vartheta_{P_{a}R_{a}Q_{a}}\left[n\right]\right)$ is the row vector containing the basis functions following the EGMP behavioral model described in (5), i.e., the *n*th row of the data matrix E. Finally, the Jacobian of the SED-GMP model is obtained taking into account all $J_{f_{\mathrm{mls}}}$ as follows.

(18) $$J=\sum_{m=0}^{M_{a}}\sum_{l=0}^{L_{a}}\sum_{s=0}^{S_{a}}\begin{array}{c} J_{f_{\mathrm{mls}}} \end{array}$$

With the Jacobian matrix, it is now possible to address the nonlinear least squares problem, for example, by using the Levenberg–Marquardt (LM) algorithm. The LM algorithm is an iterative procedure, where the vector of coefficients α is updated at every iteration *i*, as follows:

(19) $$\begin{array}{c} \alpha^{i+1}=\left(1-\gamma \right)\alpha^{i}-\gamma {\left(J^{H}J+\lambda I\right)}^{-1}J^{H}e_{\mathrm{BB}} \end{array}$$

where I is the identity matrix, γ (0<γ<1) is the learning rate, λ is the damping factor, and J is the Jacobian matrix being calculated over the error vector $e_{\mathrm{BB}}$ with respect to α.

The complete procedure for training the EGMP model is shown in Algorithm 1. The process is inspired in the DDPG algorithm, which is a popular algorithm for reinforcement learning [20]. The SED-GMP model can be described as the critic that estimates the action value, the EGMP model as the actor or policy that generates the action (supply envelope), and the Jacobian of the EGMP model as the policy gradient. In a reinforcement learning architecture, exploration noise is added to the actions to evaluate and explore the actions that are not generated by the actor. However, in the ET PA system, injecting random noise into the supply envelope is not recommended. On one hand, because the envelope random noise can modulate the output of the ET PA and degrade the overall linearity; and on the other hand, because the slew-rate of the random noise is not under control, and thus the slew-rate limitation would not be met. Therefore, in this paper, multiple shaping functions with different configurations are defined in order to perform the supply envelope exploration. For example, several SR reduced envelopes can be generated by considering different number of SR reduction steps in (4) (e.g., N= 30, 60, 90), so that the ET PA can experience the same baseband input with different supply envelopes, instead of adding noise to the output of the PA. In step 2 of Algorithm 1, $\Gamma_{1}\cdots \Gamma_{k}$ define *k* different shaping functions and generate *k* different envelopes ${\mathrm{Env}}_{gen,1}\cdots {\mathrm{Env}}_{gen,k}$ in step 5. Since the generation of SED-GMP matrix depends on the input envelope, in step 7 and 15, the SED-GMP matrix $U_{1}\cdots U_{k}$ are generated separately and concatenated row-wise to U. In steps 10 and 18, for the LM estimation of $\alpha^{\prime }$, only $U_{1}$ is used to optimize the EGMP model, targeting the first envelope generation function $\Gamma_{1}$. From the ET PA behavioral modeling point of view, the exploration process with different supply envelopes enriches the statistics of the training data and helps to create a precise SED-GMP model, which has a positive impact on the optimization process of the EGMP model. As it will be shown in the experimental results section (Section 3), thanks to the exploration process, it is possible to convergence to a global optimum, while without exploration, the training process can diverge after several iterations.
**Algorithm 1** Adaptation procedure for EGMP1:Initialize the EGMP and SED-GMP model2:Initialize several shaping functions $\Gamma_{1}\cdots \Gamma_{k}$3:Initialize weights α←{0}, β←{0}4:Generate sample signal u5:Generate envelope ${\mathrm{Env}}_{gen,1}\cdots {\mathrm{Env}}_{gen,k}$6:Generate EGMP matrix E7:Generate SED-GMP matrix $U=U_{1}\cdots U_{k}$         ▹ Row stack8:$y=y_{1}\cdots y_{k}\leftarrow trx\left(U,{\mathrm{Env}}_{gen,1}\cdots {\mathrm{Env}}_{gen,k}\right)$9:$\beta^{0}\leftarrow {\left(U^{H}U\right)}^{-1}U^{H}y$10:$\alpha^{0}\leftarrow {\left(J^{H}J+\lambda I\right)}^{-1}J^{H}\left(U_{1}\beta^{0}-U_{\mathrm{BB}}\right)$11:i←012:**repeat**13:    ${\mathrm{Env}}_{opt}\leftarrow E\alpha_{i}$14:    ${\mathrm{Env}}_{in,1\cdots k}\leftarrow {\mathrm{Env}}_{gen,1\cdots k}-{\mathrm{Env}}_{opt}$15:    Regenerate SED-GMP matrix $U=U_{1}\cdots U_{k}$16:    $y=y_{1}\cdots y_{k}\leftarrow trx\left(U,{\mathrm{Env}}_{in,1}\cdots {\mathrm{Env}}_{in,k}\right)$17:    $\beta^{\prime }\leftarrow {\left(U^{H}U\right)}^{-1}U^{H}y$18:    $\alpha^{\prime }\leftarrow \begin{array}{c} {\left(J^{H}J+\lambda I\right)}^{-1}J^{H}\left(U_{1}\beta^{i}-U_{\mathrm{BB}}\right) \end{array}$19:    $\beta^{i+1}=\gamma \beta^{\prime }+\left(1-\gamma \right)\beta^{i}$20:    $\alpha^{i+1}=\gamma \alpha^{\prime }+\left(1-\gamma \right)\alpha^{i}$21:    i←i+122:**until**$i=i_{max}$ or meet linearity requirement23:**return**$\alpha^{i-1}$

## 3. Experimental Setup and Linearization Results

The EGMP model generates an envelope optimized for linearity, which reduces the nonlinear distortion at the PA output. However, as the nonlinear distortion grows with signal bandwidth, for certain signal bandwidth configurations, the baseband I-Q DPD (e.g., based on the GMP behavioral model) is still required to compensate for the distortion caused by the PA’s nonlinear characteristic. As depicted in Figure 1, the proposed linearization strategy combines the shaping function, the envelope optimizer (based on the EGMP model), and an optional I-Q DPD. The following subsections provide a brief description of the experimental setup, as well as experimental results validating the proposed linearization strategy when considering a 60 MHz bandwidth new radio (NR) 5G signal.

### 3.1. Experimental Setup

**Figure 8:**
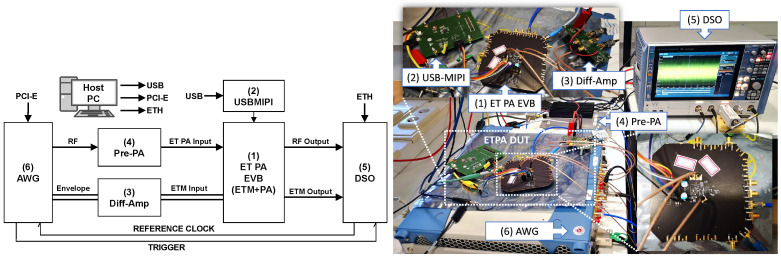
Simplified block diagram and picture of the ET PA linearization test bench. The labels are as follows: (1) ET PA EVB: HiSilicon’s ET PA evaluation board including ETM and RF ET PA; (2) USB-MIPI: FTDI USB to MIPI bridge; (3) Diff-Amp: Differential amplifier THS4508RGT from Texas Instruments; (4) Pre-PA: RF pre-amplifier ZHL-42 from Minicircuits; (5) DSO: Digital oscilloscope RTP084 from Rohde and Schwarz; (6) AWG: Arbitrary waveform generator M8190A from Keysight.

The proposed ET PA linearization approach was evaluated using a remote Matlab-controlled digital linearization test bench, as shown in Figure 8, interfacing arbitrary waveform generation (AWG M8190A from Keysight) and signal acquisition (DSO RTP084 from Rohde and Schwarz) instruments. The system-on-chip ET PA board was developed by HiSilicon for mobile applications. The envelope signal is generated by the AWG and is pre-amplified by the differential amplifier, then sent to the ETM input with a differential connection. The following tests results were obtained by operating the ET PA with a 5G NR signal of 60 MHz bandwidth and 6.8 dB of PAPR at band 7 (i.e., 2.55 GHz). The shaping function to dynamically supply the ET PA is the slew-rate reduction (SR) envelope described in Section 2.1.

### 3.2. Experimental Results with the NR-60 MHz Test Signal

This subsection shows the experimental results of applying the proposed agnostic digital linearization approach for ET PAs when operated with a 60 MHz 5G-NR QPSK modulated signal at band 7. Every data batch consists of 0.5 ms of transmitted signal, which contains 1 slot in a 30 kHz subcarrier spacing (SCS) subframe. Considering a baseband clock of 614.4 MHz (corresponding to an observation and predistortion bandwidth at baseband around 300 MHz), the number of data samples for every batch is 307,200. The maximum number of epochs per iteration for the nonlinear least squares coefficient adaptation was set to 17; this way it was possible to observe the stability of the proposed linearization method. In order to provide a fair comparison between the ELC approach in [19] (that requires measuring the supply envelope at the ETM output) with the EOPT approach proposed in this paper, the same input data with the same input power were used for testing.

**Figure 9:**
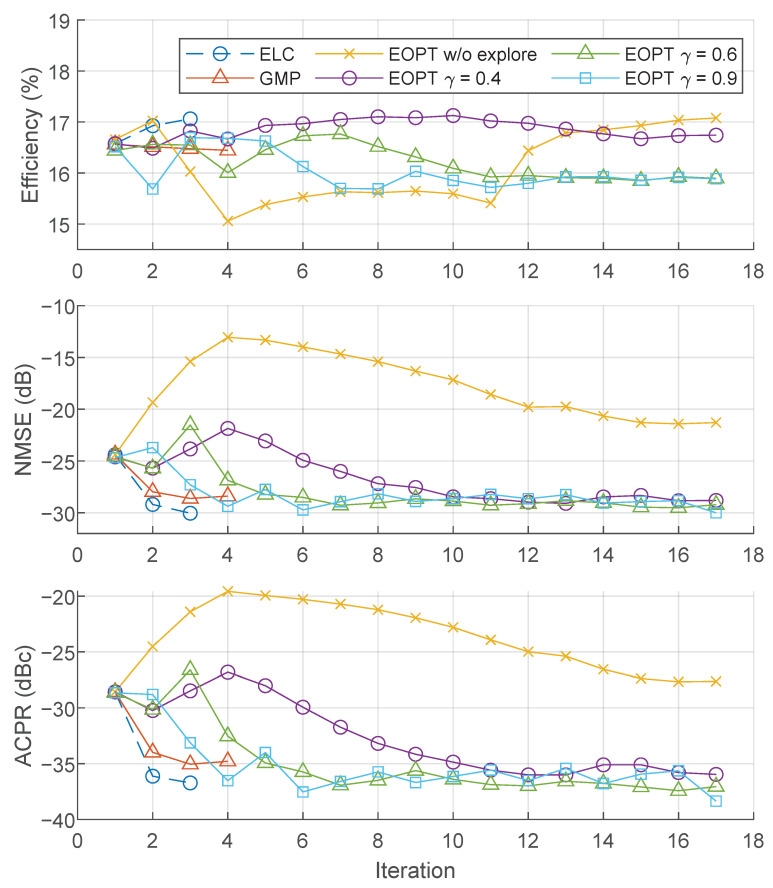
Power efficiency, NMSE, and ACPR performance per iteration of different linearization approach.

Figure 9 shows the evolution of key performance indicators such as power efficiency, NMSE, or ACPR over several training iterations of the EOPT when considering different test cases. These test cases include ELC, GMP-based I-Q DPD and EOPT linearization with and without exploration, and different learning rate values (i.e., γ in (12) and (19)) for the EOPT training process. The supply envelope used is the SR reduction shaping with steps N=30 and offset percentage OP=20%. The power efficiency can be well maintained (i.e., the efficiency is kept or improved), with ELC, GMP-based I-Q DPD or EOPT linearization with γ=0.4, and exploration. The power efficiency with EOPT, but without the envelope exploration process, experienced significant variations along the training iterations. When considering EOPT with the exploration process and a high learning rate, e.g., γ=0.6 or γ=0.9, the efficiency had a smaller variation but finally converged to a lower value compared to the initial power efficiency value. Regarding the linearity performance (in terms of NMSE and ACPR), only the EOPT without the envelope exploration process fails to improve the overall linearity. With the ELC approach, the linearity requirement of −36 dBc of ACPR is met with only two iterations of the ELC adaptation. The GMP-based I-Q DPD can improve the linearity after two training iterations, but after that it does not improve further, and the targeted ACPR cannot be met (e.g., the results in Figure 9 show up to the fourth iteration of the GMP-based I-Q DPD to evidence its limitations).

**Figure 10:**
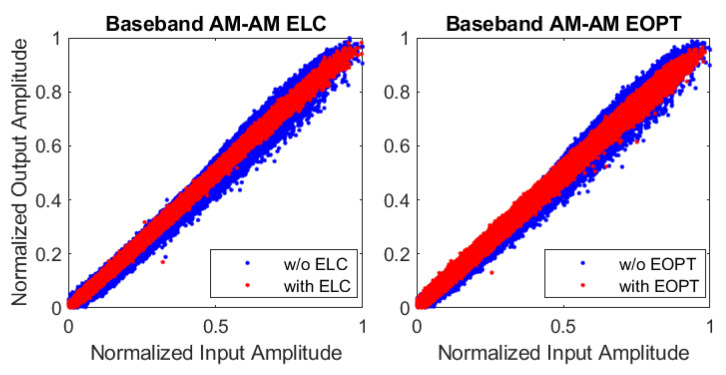
AM–AM plot of the normalized baseband input and ET PA output.

**Figure 11:**
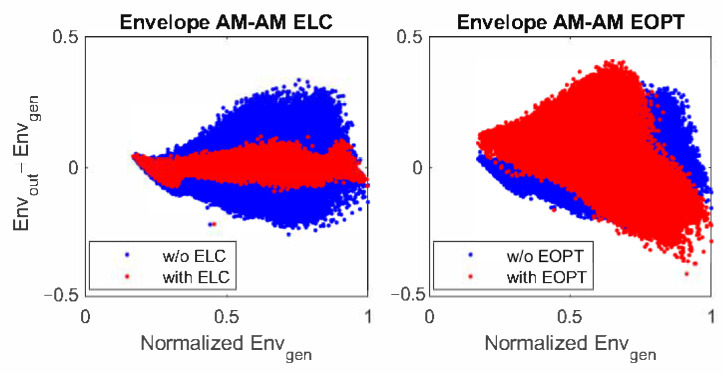
AM–AM plot of the normalized generated envelope ($Env_{gen}$) and the ETM output envelope difference (i.e., $Env_{out}-Env_{gen}$).

Figure 10 and Figure 11 show the AM–AM characteristics of the ET PA and the ETM with and without ELC and EOPT linearization, respectively. The baseband AM–AM plots are similar to each other since both linearization approaches reached the same level of linearity at the end. The AM–AM characteristic at the ETM relating the generated envelope and the difference between the ETM output and generated envelopes, evidences the difference between the ELC and the EOPT linearization approaches. In both cases, the normalized $Env_{gen}$ starts taking values from 0.2 because the OP configuration of the SR envelope is set to 20%. With the ELC linearization approach, the RF leakage is treated as the error to be canceled by using the EGMP model. Consequently, the AM–AM plot after ELC linearization shows the linearization efforts to achieve a constant flat gain. With the EOPT linearization approach, instead, since the ETM output is not available, canceling the RF leakage is not the objective. As observed in the AM–AM plot in Figure 11-right, the RF leakage is not compensated at the ETM output ($Env_{out}$) after EOPT linearization; instead, as it will be shown later, the resulting supply envelope is optimized for linearity at the ET PA output.

**Figure 12:**
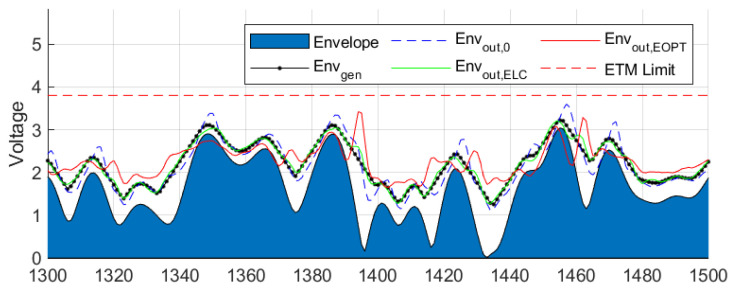
Time domain plots of different supply envelopes without and with different linearization approaches.

Time domain plots of different supply envelopes are depicted in Figure 12. As observed, $Env_{out,0}$, which is the envelope at the ETM output when no linearization is applied, contains high-frequency leakage in comparison to the generated envelope $Env_{gen}$. Most of the RF leakage can be compensated with the ELC linearization approach, as observed in $Env_{out,ELC}$ in Figure 12. Instead, $Env_{out,EOPT}$ (i.e., the supply envelope after EOPT linearization) does not show any RF leakage compensation. However, the resulting supply envelope after EOPT linearization is optimized for linearity at the ET PA output. In any case, neither $Env_{out,ELC}$ nor $Env_{out,EOPT}$ envelopes exceed the ETM’s linear amplification limitation (around 3.8 V).

**Figure 13:**
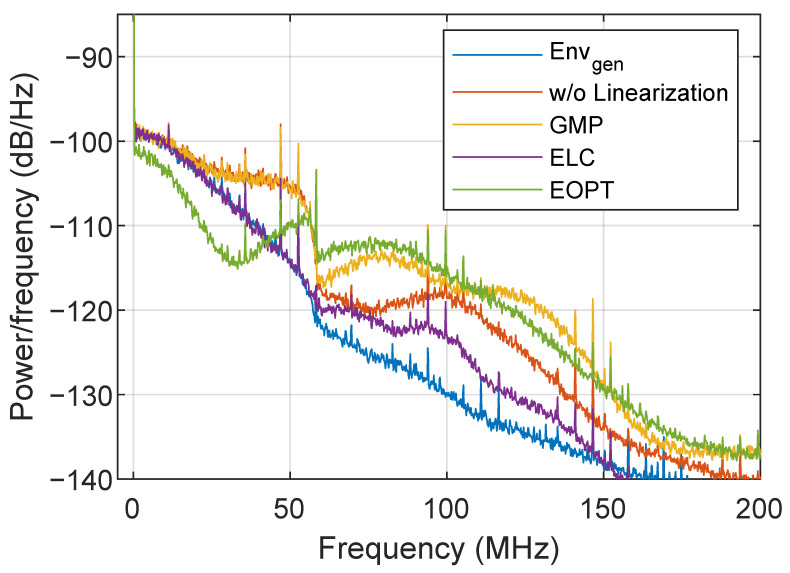
Spectra of different supply envelopes without and with different linearization approaches.

**Figure 14:**
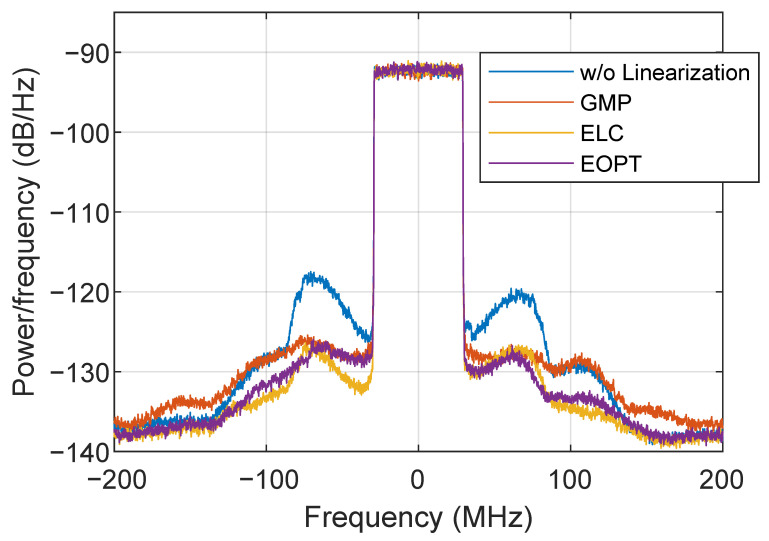
Spectra of the ET PA output signal without and with different linearization approaches.

Figure 13 shows the spectra of different supply envelopes before and after applying different linearization methods. By considering a vertical margin of 40 dB, the generated SR envelope ($Env_{gen}$) has around 100 MHz bandwidth. The supply envelope at the ETM output without linearization has around 140 MHz bandwidth, i.e., the RF leakage has a bandwidth of around 140 MHz. Consequently, both the ELC or EOPT linearization approaches will generate an envelope optimization signal mostly within this bandwidth. After applying GMP-based I-Q DPD, the ETM output spectrum shows a spectral regrowth above 60 MHz. Despite the fact that the GMP-based I-Q DPD generates an I-Q predistorted signal targeting the linearization of the ET PA, part of the linearized RF signal is leaked to the ETM output and combined with the supply envelope, degrading again the linearity at the ET PA output. After ELC linearization, the spectrum of the ETM output is very close to the spectrum of the generated envelope $Env_{gen}$, although there are some variations from 60 to 150 MHz. As observed, the purpose of EOPT linearization is not canceling the RF leakage, since the spectrum of the ETM output does not resemble the generated envelope. However, as shown in Figure 14, thanks to the EOPT linearization, the linearity requirements are met at the ET PA output.

**Figure 15:**
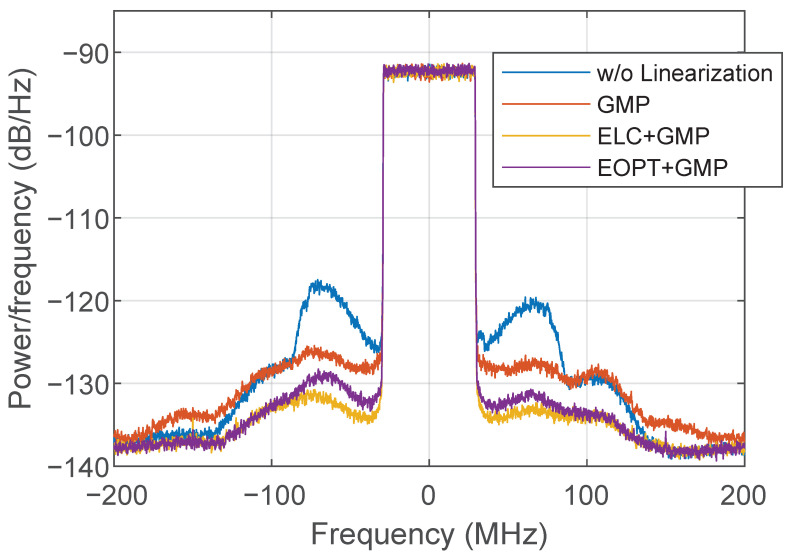
Spectra of the ET PA output signal when combining the use of different envelope linearization approaches and I-Q DPD.

Figure 14 presents several ET PA output spectra when considering different linearization approaches. Without applying any linearization, the output spectrum shows strong leakage at its adjacent channels, with a significant spectral regrowth at around 60 MHz away from the center frequency. This specific spectral regrowth can be somehow compensated by the GMP-based I-Q DPD (including cross memory and high-order terms). However, as shown in Figure 14, the ET PA output spectra after GMP-based I-Q DPD fails to further compensate for the distortion at higher frequencies. Instead, when considering the linearization approaches at the supply envelope path (i.e., the ELC or EOPT), the overall spectral regrowth is mitigated, despite the fact that the peaks appearing at 60 MHz can still be appreciated. Although both envelope linearization approaches are enough to meet the linearity specifications (i.e., ACPR <−36 dBc), to further improve the overall ET PA linearity, these can be combined with the conventional I-Q DPD linearization. Figure 15 shows ET PA output spectra when both ELC and EOPT envelope linearization techniques are combined with GMP-based I-Q DPD (i.e., ELC+GMP and EOPT+GMP, respectively).

Table 1 provides a comparison of different linearization approaches. As observed, without any kind of linearization, the ET PA output presents −24.39 dB of NMSE and −28.57 dBc of ACPR, with a power efficiency of 16.58%. By considering the GMP-based I-Q DPD with 128 complex-valued coefficients, the ACPR requirement of −36 dBc cannot simply be met. However, with the ELC or the proposed EOPT linearization, the ACPR requirements can be met with 84 real-valued coefficients. Regarding the computational resources, each GMP coefficient results in a complex-to-real and a complex-to-complex multiplication; while each ELC/EOPT coefficient only uses two real-to-real multiplications. The actual number of multiplication depends on the implementation architecture. For example, by considering three real multipliers for the complex-to-complex multiplication, then 128 GMP coefficients end up using 5×128=640 multipliers; while 84 ELC/EOPT coefficients only require 2×84=168 multipliers. It is important to note that both ELC and EOPT approaches use the same EGMP basis functions with maximum memory depth of 20 taps and maximum power order of 6. The only difference between both approaches is the procedure to extract the EGMP coefficients (i.e., the ETM output is not available to extract the EGMP coefficients in the EOPT approach). Both approaches show similar performance; however, the ELC has slightly better NMSE and power efficiency while the EOPT has slightly better ACPR performance. Finally, by combining the use of envelope optimization and I-Q DPD, the linearity can be further improved. As shown in Table 1, despite the fact that the ELC+GMP combination presents the best linearity performance with −33 dB of NMSE and −40.32 dBc of ACPR, the EOPT+GMP alternative shows very competitive results not only in terms of linearity but also in terms of power efficiency. In addition, since the proposed EOPT approach is agnostic on the ETM behavior, the cost of an extra ADC to monitor the ETM output is saved.

## 4. Conclusions

A new envelope linearization system for ET PAs is presented in this paper. The proposed EOPT linearization approach uses an EGMP behavioral model to optimize the supply envelope in order to meet the linearity requirements at the ET PA output. Despite that the architecture is similar to the ELC approach, since the output of the ETM is not available, the EOPT is agnostic on the ETM behavior. Consequently, a new procedure, based on the reinforcement learning DDPG algorithm, is used to extract the coefficients of the EGMP model. The proposed identification method exploits the SED-GMP ET PA behavioral model to estimate the EGMP coefficients solving a nonlinear least squares problem by means of the LM algorithm.

The reported experimental results illustrate how the EOPT linearization approach can outperform the conventional GMP-based I-Q DPD linearization (up to 2 dB of ACPR improvement). In addition, the EOPT method can achieve similar linearization performance to the ELC approach (i.e., ACPR close to −37 dBc) with the same number of parameters (84 coefficients) and similar power efficiency (close to 17%). However, the EOPT method is agnostic of the EMT behavior and it does not need monitoring the ETM output, which saves the cost of an extra ADC. Finally, as reported in the experimental results section, the proposed envelope optimization technique can be properly combined with I-Q DPD to improve the ACPR figure by 4 dB.

## Figures and Tables

**Table 1:** ET PA experimental results with NR-60 MHz OFDM-like signal at band 7.

Linearization	#	NMSE	ACPR	Power	Eff.
Approach	Coeffs.	(dB)	(dBc)	(dBm)	(%)
w/o DPD	N/A	−24.39	−28.57	27.21	16.58
GMP	128	−28.39	−34.79	27.17	16.44
ELC	84	−30.04	−36.73	27.26	17.06
EOPT	84	−29.27	−36.93	27.26	16.76
ELC+GMP	84 + 64	−33.07	−40.32	27.23	16.36
EOPT+GMP	84 + 64	−32.06	−38.36	27.34	16.76

## Data Availability

The data presented in this study are available on reasonable request from the corresponding author.
